# Establishment of an indirect ELISA for *Mycobacterium tuberculosis* MTB39A protein antibody

**DOI:** 10.1007/s00253-023-12715-w

**Published:** 2023-08-19

**Authors:** Pu Wang, Yurong Cai, Gang Zhang, Lingling Jiang, Yong Li

**Affiliations:** 1 School of Life Sciences, Ningxia University, Yinchuan, China; 2 Key Laboratory of Ministry of Education for Conservation and Utilization of Special Biological Resources in Western China, Ningxia University, Yinchuan, China

**Keywords:** *Mycobacterium tuberculosis*, MTB39A, Indirect ELISA, Prokaryotic expression

## Abstract

**Abstract:**

The MTB39A protein is a member of the unique *Mycobacterium tuberculosis* (*MTB*) PE/PPE protein family and is the main candidate for tuberculosis (TB) diagnosis. The aim of this study was to establish a novel indirect ELISA (iELISA) method that uses antibodies against *MTB*. The MTB39A gene sequence was synthesized according to the MTB39A nucleotide sequence of the *MTB* H37Rv strain (GenBank accession number: NC_000962.3) and cloned into the pET28a( +) vector. After correct sequencing, it was transferred to *Escherichia coli* BL21 (DE3) receptor cells for expression and purification, and the purified recombinant protein was identified by SDS-PAGE and western blotting. The purified MTB39A protein was used as the capture antibody, and a rabbit polyclonal antibody against the *MTB* MTB39A protein was used as the detection antibody to establish an indirect ELISA method. The ELISA conditions were optimized, and the optimal coating concentration of the MTB39A antigen was determined to be 0.5 μg/mL. The optimal dilution of MTB39A rabbit polyclonal antibody was 1:4096, and the optimal dilution of HRP-goat anti-rabbit IgG was 1:4000. The results showed that this indirect ELISA method has high sensitivity, specificity and efficacy for MTB39A protein detection. Moreover, this indirect ELISA method has optimal stability and can be used for the initial detection of *MTB* antibodies in clinical human and bovine serum samples. The establishment of this assay provides a new method for the rapid diagnosis of *MTB* and technical support for the prevention and control of tuberculosis.

**Key points:**

*• MTB MTB39A protein was expressed in a prokaryotic expression system.*

*• Rabbit polyclonal antibody against MTB39A was prepared.*

*• To establish an iELISA based on the MTB39A protein for the detection of MTB antibodies.*

## Introduction

*Mycobacterium tuberculosis* (*MTB*) is the pathogen that causes tuberculosis (TB), and it is also the most important cause of death from single microbial infection. The disease mainly infects the lungs (pulmonary tuberculosis), but it can also infect other areas of the body (extrapulmonary tuberculosis). According to data published by the World Health Organization in 2022 (WHO 2022), at present, 1/4 of the world’s people are currently infected with *Mycobacterium tuberculosis*, approximately 10 million people suffer from TB every year, TB caused by *Mycobacterium bovis* accounts for 5% of all TB cases, and approximately 1.2 million people die from TB (Roos et al. 2018). It has caused huge economic losses to the human and cattle breeding industry in many countries around the world, making the prevention and control of tuberculosis of utmost importance. Early diagnosis can decrease the transmission of TB, which is greatly important for the prevention and control of TB (Burki 2010).

At present, the early diagnosis methods of TB include chest X-ray, bacteriological tests, purified protein derivative (PPD) skin tests, and nucleic acid amplification molecular biology detection, which are limited in clinical use due to the need for specialized instrumentation or long testing cycles (Correia-Neves et al. 2019; Sanchez-Cabral et al. 2020). In contrast, ELISA is widely used for the detection of various pathogenic bacteria due to its high specificity, sensitivity, reproducibility, and applicability for large sample volumes. Therefore, the search for MTB antigens with excellent sensitivity and specificity that can be used in the serological detection of TB remains an important goal for early detection and diagnosis.

To date, the diagnostic markers for *MTB* include the Ag85 complex (Barbier et al. 2023), ESAT-6 (Dewi et al. 2019; Zhang et al. 2022), CFP10 (Seele et al. 2023), MPT64 (Wang et al. 2020), and PPE17. Among them, the vaccine MVA85A with Ag85A as the antigen was found to be ineffective in enhancing the protective effect of BCG-immunized infants against *MTB* infection (Tameris et al. 2013). The positive detection rate of ESAT-6 and CFP10 for TB is only 80%, and it is hard to distinguish latent infections from active TB infections (Dosanjh et al. 2011). The MTB39A (PPE 18) protein, which belongs to the PE/PPE protein family (Hakim and Yang 2020; Ottenhoff 2020; Ullah et al. 2020), has 99% homology with the MB97 gene in *Mycobacterium bovis AF1228/2122* and may be a potential diagnostic marker in humans and bovines due to its high specificity and optimal immunogenicity (Dolasia et al. 2021).

Therefore, this study established a rapid, effective, and high-throughput indirect ELISA detection method using purified *MTB* MTB39A recombinant protein as the coating antigen, providing a new method for the detection of *MTB* infection and evaluation of vaccine immune efficacy.

## Materials and methods

### Strain and serum samples

The prokaryotic expression vector pET28a( +) (Invitrogen, USA) and *Escherichia coli* BL21 (DE3) strain (Invitrogen, USA) were preserved in our laboratory. Serum from patients positive and negative for TB was provided by the Fourth People’s Hospital of Yinchuan, Ningxia. New Zealand white rabbits (male, 2.5 kg) were purchased from Ningxia Medical University (certificate number: IACUC-NYLAC-20).

### Construction of the MTB39A gene recombinant plasmid

The MTB39A gene (Nanjing Kingsray Biotechnology Co., Ltd.) was designed by selecting the corresponding gene sequence of the MTB39A protein from the *MTB* H37Rv strain (GenBank accession number: NC_000962.3) and the His tag sequence as the purification site. The pET28a( +) vector was ligated with the MTB39A gene using *Nde*I and *Hin*dIII endonucleases. The plasmid was extracted, digested and detected by agarose gel electrophoresis and verified by sequencing (Jilin Kumi Co., Ltd.), and the sequence of the target gene was compared with the sequencing result sequence by Snapgene 3.2.1 software.

### Identification of the optimal conditions for induction of recombinant MTB39A protein expression in *E. coli.*

The identified recombinant plasmid was transferred into *E. coli* BL21 (DE3) receptor cells for the induction of expression. The induction (37 °C, 180 rpm) was carried out at 0.25 mM, 0.5 mM, and 1 mM concentrations of IPTG for 3 h and 16 h and incubated at different temperatures of 20 °C and 16 °C to determine the optimal induction expression conditions. The induced bacterial broth was centrifuged, and the supernatant and precipitate were collected separately, added to protein loading buffer, and subjected to SDS-PAGE electrophoresis in a boiling water bath for 10 min, as well as Coomassie brilliant blue (CBB).

### Purification and western blot identification of the recombinant MTB39A protein

After determining the optimal conditions for expression induction, the bacteria were cultured in large numbers and sonicated to kill the bacteria, and the supernatant and precipitate after sonication were collected to analyze the expression of the recombinant protein. MTB39A protein was purified using Ni^2+^ affinity chromatography, eluted with elution buffer (20 mmol/L Tris–HCL, 500 mmol/L imidazole), and finally, the target protein was gradient-replicated at 4 °C at 6 M, 4 M, 2 M, and 0 M. The purified protein concentrations were quantified using the BCA Protein Assay Kit (KeyGEN, Jiangsu, China). The obtained inclusion bodies were identified by SDS-PAGE and western blotting with the purified proteins.

### Recombinant MTB39A protein activity assay

The purified MTB39A protein was subjected to SDS-PAGE and blotted onto 0.22-μm nitrocellulose membranes using a Bio-Rad Mini Trans-Blot Cell (Bio-Rad) for western blotting. Closure was performed using 5% skim milk powder, followed by overnight incubation at 4 °C with human healthy/TB-positive, bovine healthy/TB-positive sera diluted to 1:10, incubated with HRP-coupled anti-human/bovine, washed three times with TBST (20 mM Tris, 150 mM NaCl, 0.2% Tween 20), and finally with ECL luminescence reagent (Abclone, China) and placed in an image acquisition instrument for imaging (GE Amersham Imager600, USA). Fourteen human TB-positive serum samples, five human TB-negative serum samples, five bovine TB-positive serum samples, and one bovine TB-negative serum samples from the Fourth People’s Hospital of Ningxia Hui Autonomous Region (China) were determined to be TB-positive by positive sputum smears and lung pathology cuts.

### Preparation of MTB39A protein polyclonal antibody

The MTB39A protein was diluted to a concentration of 1 mg/mL. The protein was subcutaneously injected into the backs of New Zealand rabbits at 0, 10, 20, and 30 days. Each immunization was performed with 1 mL of adjuvant and 1 mg/mL of antigen, which was emulsified with an equal volume of Freund’s adjuvant. Freund’s complete adjuvant was used for the first immunization, and Freund’s incomplete adjuvant was used for the other three booster immunizations.

Blood was collected from the ear margin vein of each rabbit before immunization at 0 day. Serum was isolated and used as a negative control. On the 37th day postimmunization, blood was collected from the heart of each experimental rabbit, and the whole blood samples were placed at 37 °C for 1 h and centrifuged at 4000 rpm for 10 min at 4 °C. The separated serum was stored at − 80 °C. The antiserum was purified using a Protein A column.

### Establishment of an indirect ELISA based on the recombinant MTB39A protein antibody

The purified MTB39A protein was diluted with coating solution at concentrations of 0.25 μg/mL, 0.5 μg/mL, 1 μg/mL, 1.5 μg/mL, 2 μg/mL, and 2.5 μg/mL per well in 96-well ELISA plates at 100 µL/well and incubated overnight at 4 °C. Five percent bovine serum albumin (BSA) was used as the blocking solution for 30 min, 60 min, 90 min, 120 min, 60 min, 90 min, and 120 min. MTB39A rabbit polyclonal antibody was used as the primary antibody (100 µL/well) and diluted 1:2^7^, 1:2^8^, 1:2^9^, 1:2^10^, 1:2^11^, 1:2^12^, 1:2^13^, and 1:2^14^, and the negative control was normal rabbit serum. The primary antibody was incubated at 37 °C for 1 h. PBST was used to wash the plate, and the diluted (1:1000, 1:2000, 1:4000, 1:8000) secondary antibody HRP-goat anti-rabbit IgG (Proteintech) was added to each well. TMB was used for color development, and 2 mol/L H_2_SO_4_ termination solution was added to determine the OD450 nm value.

The sample P/N values (P represents the positive serum OD450 nm value, N represents the negative serum OD450 nm value) of each sample were used to determine the optimal antigen coating concentration, primary antibody dilution, enzyme-labeled secondary antibody dilution, and incubation time.

### Determination of the threshold value

The established indirect ELISA was used to determine the OD450 nm values of 20 negative serum samples, and the mean OD450 nm value (X) and standard deviation (SD) of these samples were calculated. When the OD450 nm value of the sample was greater than X + 3 SD, the sample was considered positive; when the OD450 nm value was less than X + 2 SD, it was considered negative; and when it was between the two, it was considered suspected.

### Sensitivity and specificity test

The sensitivity of the established and optimized indirect ELISA was verified by measuring the absorbance value at OD450 nm and multiplying it by the MTB39A rabbit multiple antibody dilution. Next, the specificity of the MTB39A rabbit polyclonal antibody was verified by using the MTB39A protein, *Salmonella typhimurium* 14,208, *Micrococcus garciniae*, *Escherichia coli* 83,922, *Escherichia coli* 83,905, or *Escherichia coli* 83,684 as the capture antibody and the MTB39A rabbit polyclonal antibody as the detection antibody.

### Intrabatch and interbatch replicate assays

Using the established indirect ELISA, the rabbit MTB39A polyclonal antibody and the negative antibody were tested in 3 batches and 3 interbatches to calculate the coefficient of variation, thus assessing their reproducibility.

### Detection of clinical samples

Thirty-six human TB-positive serum samples and 21 human TB-negative serum samples (serum were gifted by Ningxia No. 4 People’s Hospital) were assessed at an OD450 nm using an established ELISA method. All serum samples with OD450 nm values above 0.282 were considered positive for MTB39A antibodies, those with values less than or equal to 0.257 were recognized as negative for MTB39A antibodies, and samples with values between 0.257 and 0.282 were recognized as suspected.

## Result

### Construction and validation of the recombinant expression vector pET28a-MTB39A

**Fig. 1:**
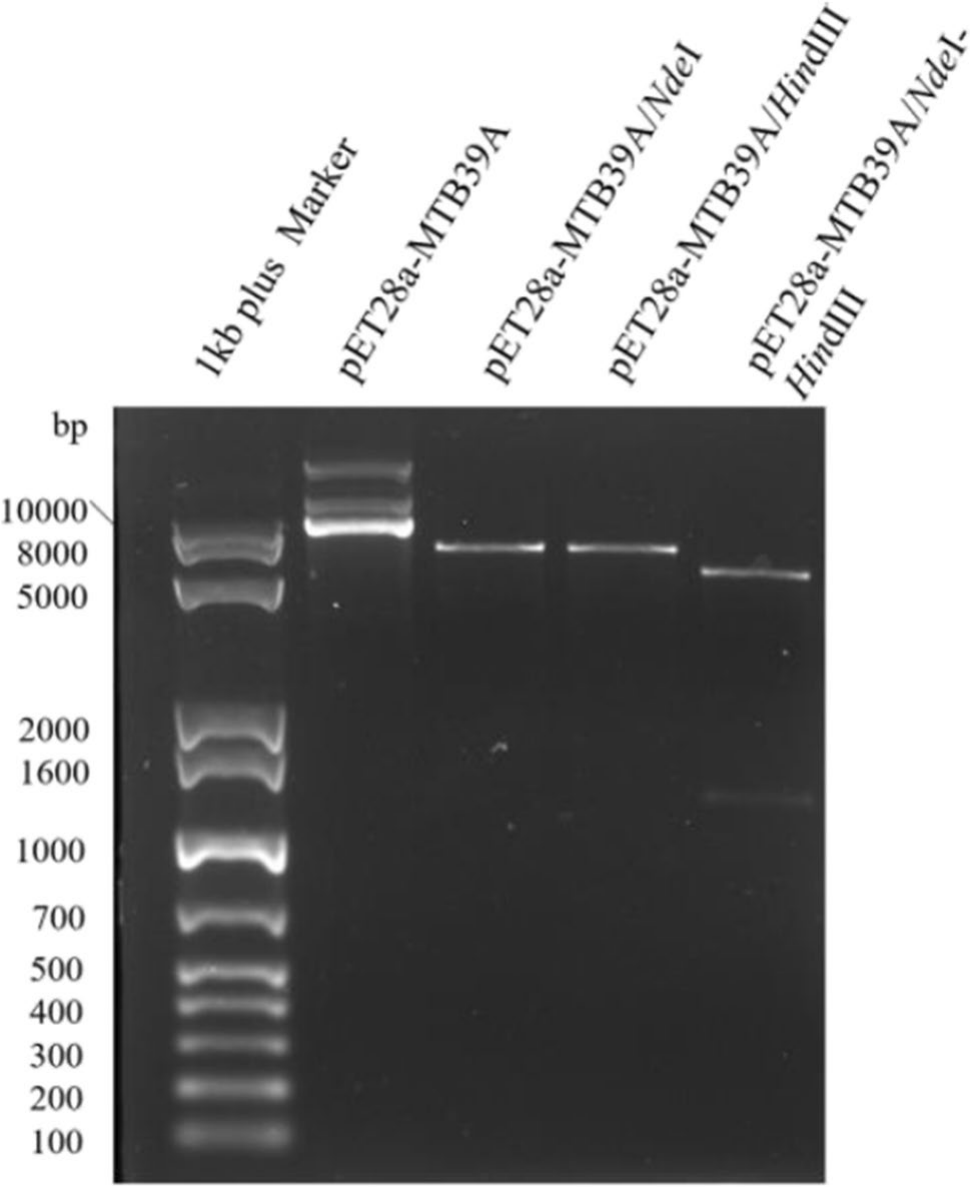
Identification of recombinant plasmids by enzyme digestion

The MTB39A gene was cloned into the pET28a( +) vector through *Nde*I and *Hin*dIII endonuclease, and the positive clone with correct digestion and sequencing was named pET28a-MTB39A. The gel electrophoresis results are shown in Fig. 1. The samples from left to right are 1 kb plus marker, pET28a-MTB39A, pET28a-MTB39A/*Nde*I (6328 bp), pET28a-MTB39A *Hin*dIII (6328 bp), and pET28a-MTB39A/*Nde*I + *Hin*dIII (5122 bp, 1206 bp). The size of the pET28a-MTB39A recombinant plasmid matches expectations.

### Purification and identification of the recombinant MTB39A protein

**Fig. 2:**
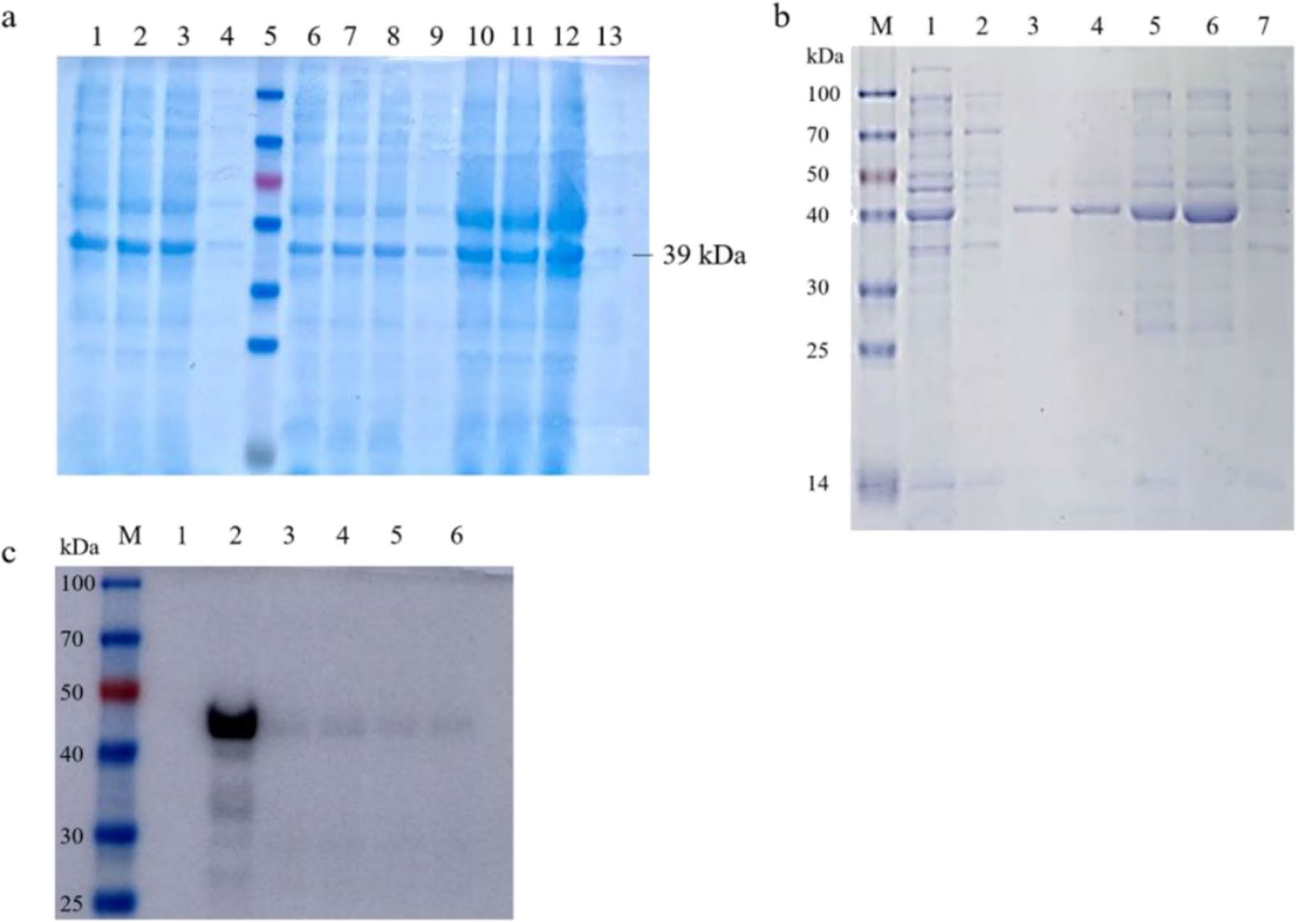
Recombinant MTB39A protein expression, purification, and identification. **a** 1: 0.25 mM IPTG, 16 °C, 16 h; 2: 0.5 mM IPTG, 16 °C, 16 h; 3: 1 mM IPTG, 16 °C, 16 h; 4: 0 mM IPTG, 20 °C, 16 h; 5: marker (14–100 kDa); 6: 0.25 mM IPTG, 20 °C, 16 h; 7: 0.5 mM IPTG, 20 °C, 16 h; 8: 1 mM IPTG, 20 °C, 16 h; 9: 0 mM IPTG, 20 °C, 16 h; 10: 0.25 mM IPTG, 37 °C, 3 h; 11: 0.5 mM IPTG, 37 °C, 3 h; 12: 1 mM IPTG, 37 °C, 3 h; 13: 0 mM IPTG, 37 °C, 3 h; **b** 1: 1 mM IPTG-induced expression and precipitation; 2: supernatant induced by 1 mM IPTG; 3–4: purified protein; 5–6: inclusion body protein crude products; 7: inclusion body protein crude product supernatant. **c** 1: inclusion body protein crude product supernatant; 2: inclusion body protein crude products; 3–6: purified protein

The identified recombinant plasmid was transferred into *E. coli* BL21 (DE3) receptor cells for the induction of expression. The induction (37 °C, 180 rpm) was carried out at 0.25 mM, 0.5 mM, and 1 mM concentrations of IPTG for 3 h and 16 h at both 20 °C and 16 °C to determine the optimal induction expression conditions. The induced bacterial broth was centrifuged, and the supernatant and precipitate were harvested separately. Protein loading buffer was added, and SDS-PAGE electrophoresis was performed in a boiling water bath for 10 min, as well as Coomassie brilliant blue (CBB) staining. Figure 2a shows that the optimal inducible expression conditions were 1 mM IPTG, 37 °C, and 3 h. After determining the best inducible expression conditions, the bacterium was cultured and killed in large quantities, and the inclusion body protein was prepared and purified. The obtained inclusion bodies and purified eluate were subjected to SDS-PAGE electrophoresis and detection of His-tagged protein. The molecular weight of the recombinant MTB39A protein was 39 kDa, as shown in Fig. 2b, and the molecular weight was consistent with the predicted size. The obtained protein was identified as MTB39A.

### Protein activity assay

**Fig. 3:**
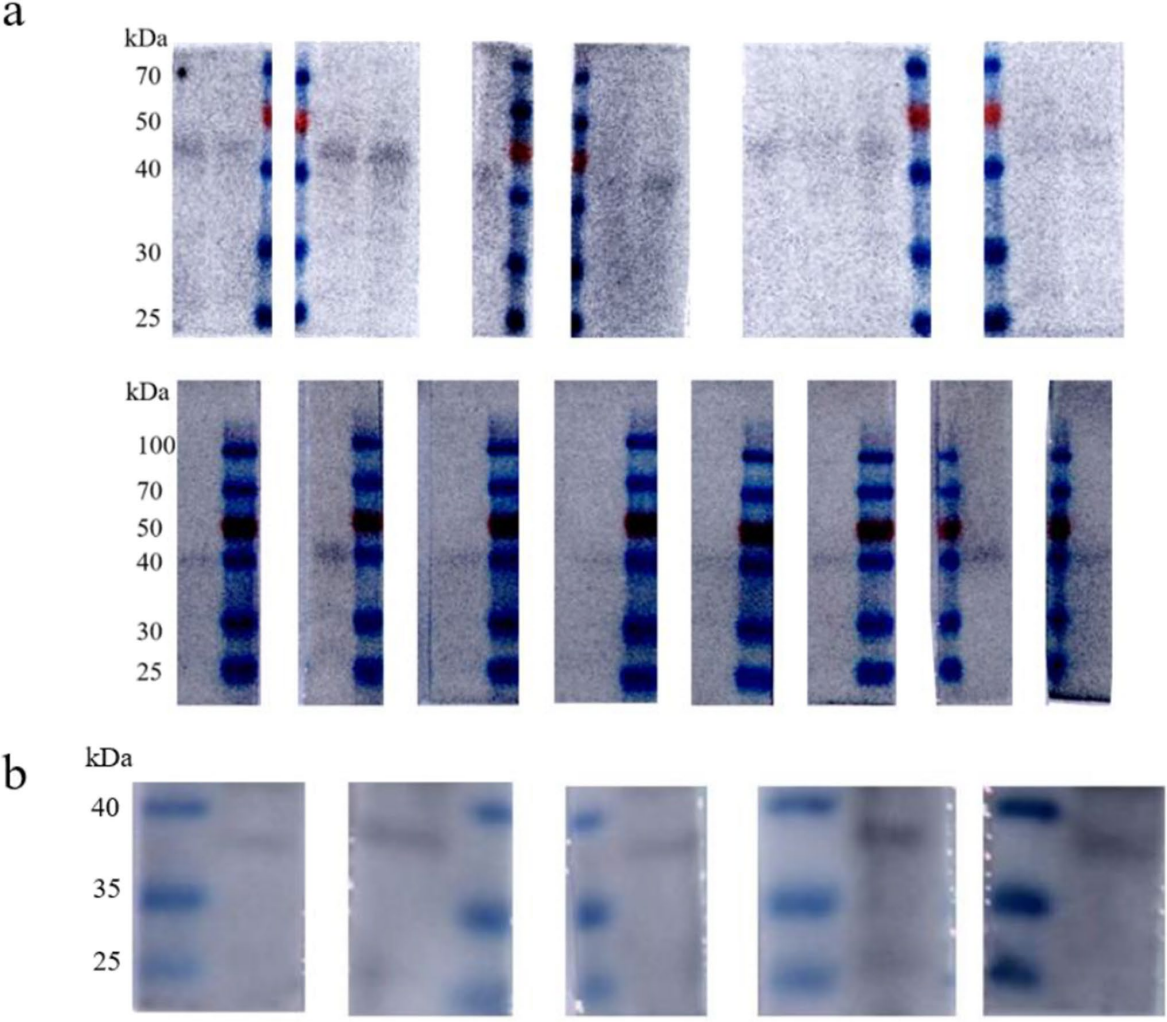
MTB39A protein activity was detected in human/bovine TB-positive serum. **a** Human tuberculosis-positive serum; **b** bovine tuberculosis-positive serum

**Fig. 4:**
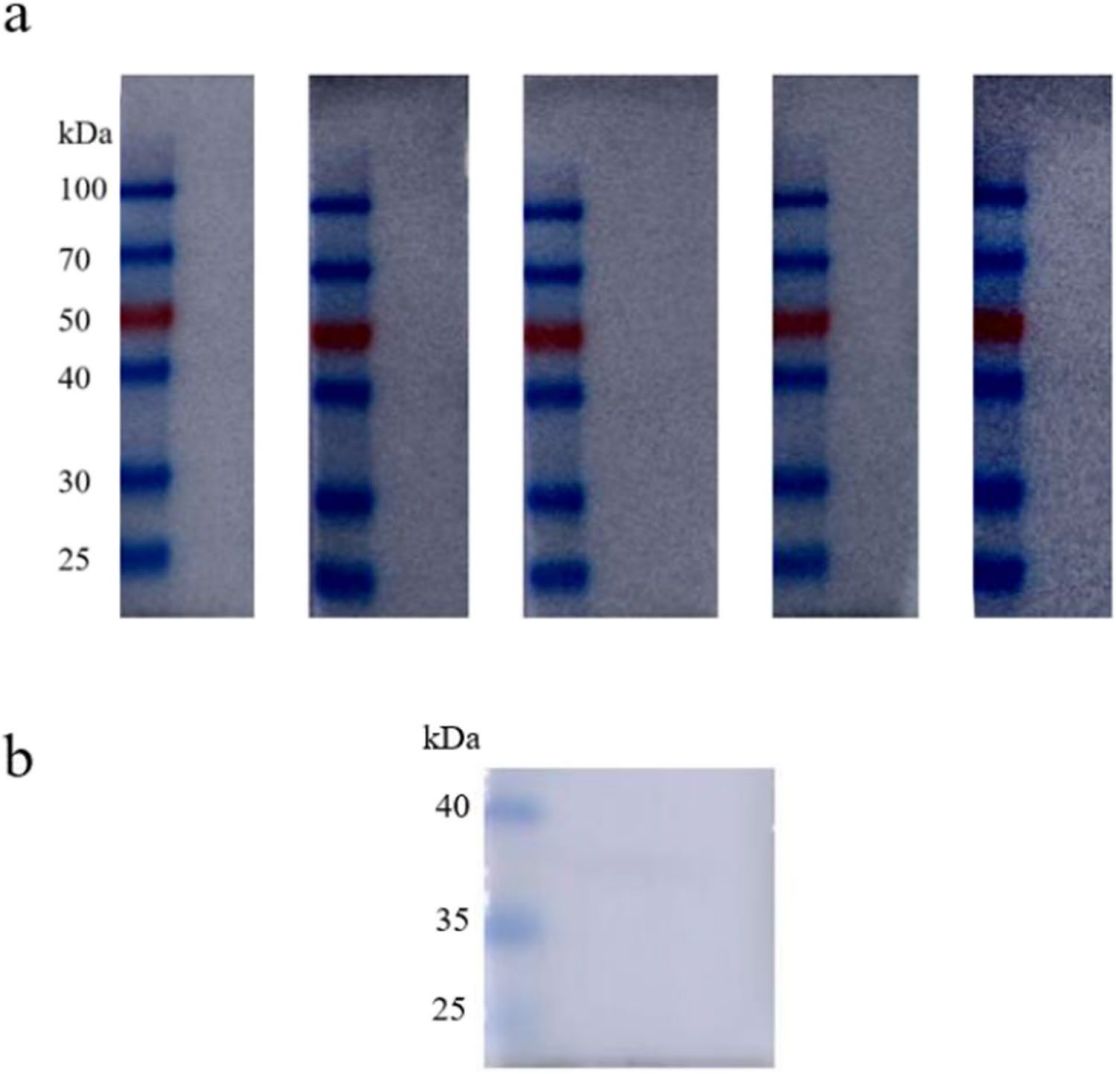
Human/bovine TB-negative serum was used to detect MTB39A protein activity. **a** Healthy human serum; **b** healthy bovine serum

The activity of the obtained MTB39A purified protein with inclusion bodies was tested using 14 human TB-positive patient serum samples, 5 healthy human serum samples, 5 bovine TB-positive serum samples, and 1 healthy bovine serum sample. As shown in Fig. 3, the MTB39A protein exhibited detectable activity against all TB-positive sera but not against healthy sera (Fig. 4). It was concluded that all of the obtained MTB39A proteins were suitable for diagnosing TB.

### Establishment of an indirect ELISA method based on the MTB39A protein polyclonal antibody

**Fig. 5:**
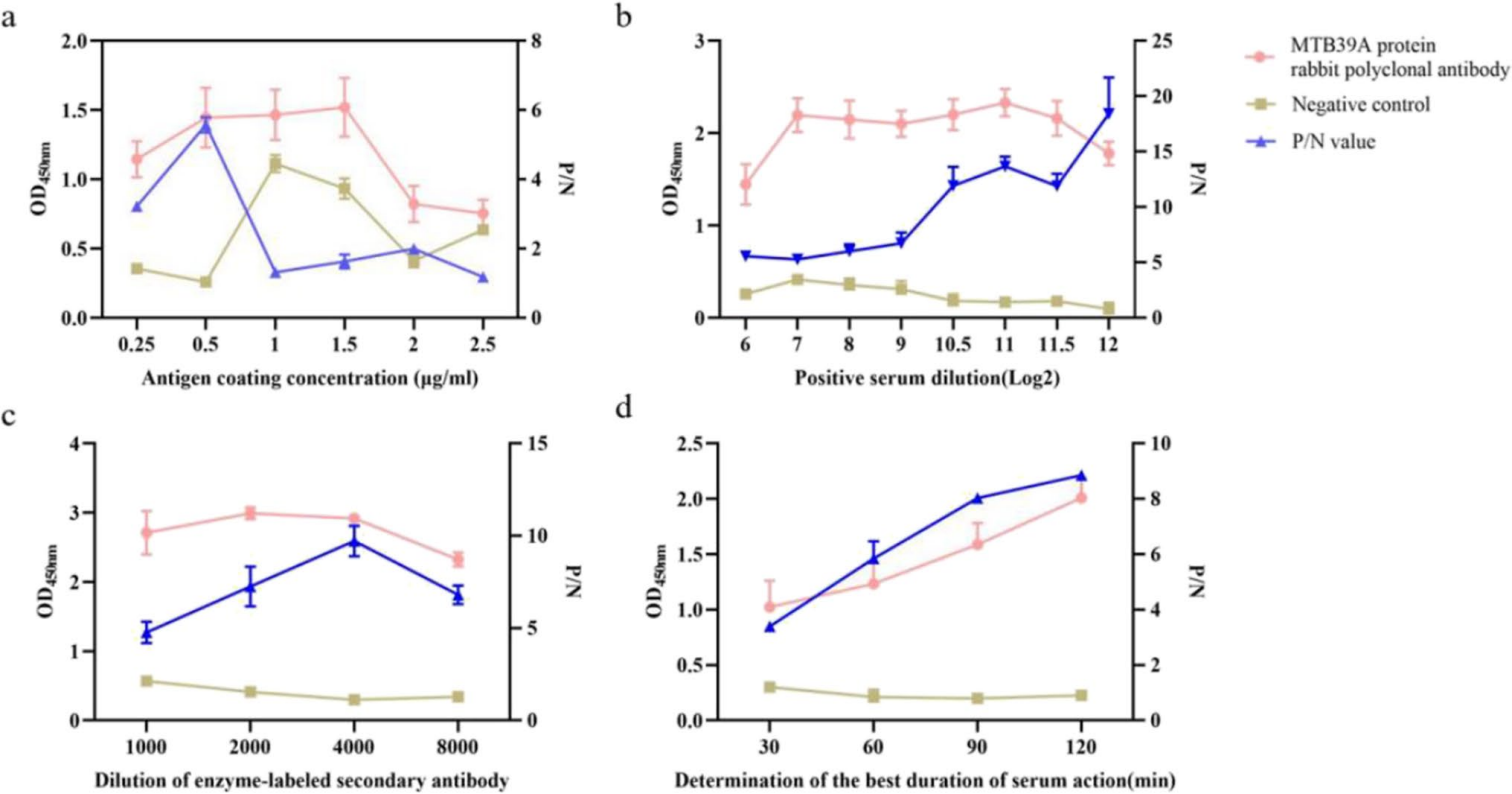
Optimization of the indirect MTB39A protein antibody ELISA conditions. **a** Optimization results of the antigen coating concentration; **b** optimization results of the suitable dilution ratio of MTB39A polyclonal antibody; **c** optimization results of the suitable dilution ratio of goat anti-rabbit enzyme-labeled antibody; **d** optimization results of serum incubation time

The MTB39A protein was used as the capture antibody, and the rabbit polyclonal antibody against the MTB39A protein was serially diluted and then subjected to indirect ELISA. The results showed that the MTB39A protein coating concentration was 0.5 μg/mL, and the rabbit polyclonal antibody against MTB39A had an OD450 nm of 1.45 and the negative control OD450 nm of 0.26, with a maximum P/N value of 5.58. Therefore, the optimal coating concentration of the MTB39A protein was determined to be 0.5 μg/mL (Fig. 5a). When the dilution of MTB39A rabbit polyclonal antibody was 2^12^, i.e., the rabbit polyclonal antibody dilution ratio was 1:4096, the OD450 nm and P/N value were the largest, and the optimal dilution of MTB39A rabbit polyclonal antibody was determined to be 1:4096 (Fig. 5b). The dilution of goat anti-rabbit enzyme-labeled antibody was optimized, and HRP-labeled goat anti-rabbit IgG was diluted at 1:4000 with the maximum P/N value (Fig. 5c). Based on the optimal working antigen concentrations, antibody and enzyme-labeled secondary antibody, the incubation time with 5% BSA was optimized, and the OD450 nm and P/N values were maximized when the 5% BSA incubation time was 120 min, so the most suitable incubation time for the MTB39A protein rabbit polyclonal antibody indirect ELISA was determined to be 120 min at 37 °C (Fig. 5d).

### Critical value determination for the indirect ELISA

Using the established indirect ELISA, the OD450 nm values of 20 negative antibodies were measured (Table 1), and the mean OD450 nm value (X) and standard deviation (SD) of these samples were calculated. The samples were considered positive when the OD450 nm value > X + 3 SD, negative when the OD450 nm value < X + 2 SD, and suspected when they were in between.

**Table 1:** OD450 nm determination of 20 negative serum samples

Number	OD_450nm_
1	0.242
2	0.239
3	0.255
4	0.240
5	0.239
6	0.237
7	0.186
8	0.193
9	0.189
10	0.185
11	0.191
12	0.197
13	0.186
14	0.191
15	0.214
16	0.196
17	0.186
18	0.186
19	0.193
20	0.195

**Fig. 6:**
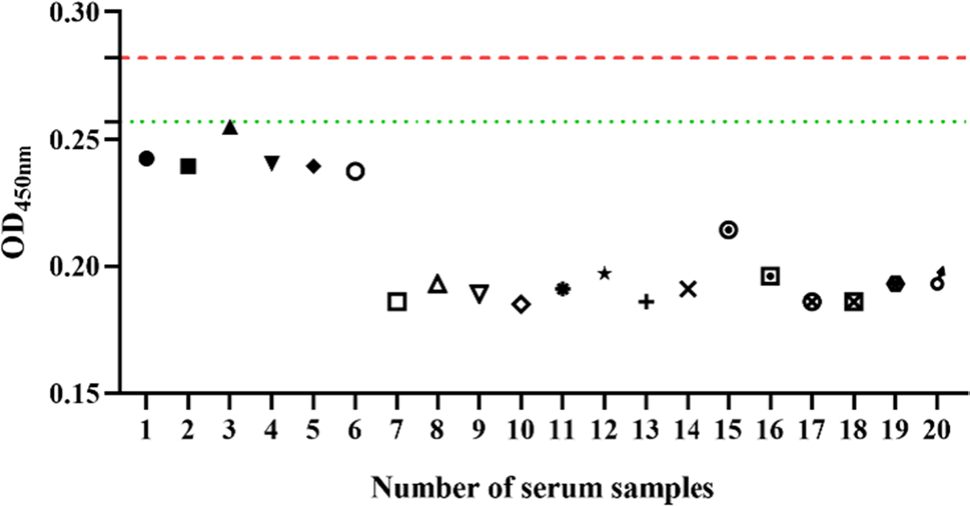
Determination of critical values

By statistical analysis, the overall mean value of the negative control was 0.207, and the standard deviation (SD) was 0.025, so the positive threshold value was 0.282, and the negative threshold value was 0.257 (Fig. 6).

### Specificity and sensitivity of the indirect ELISA

**Fig. 7:**
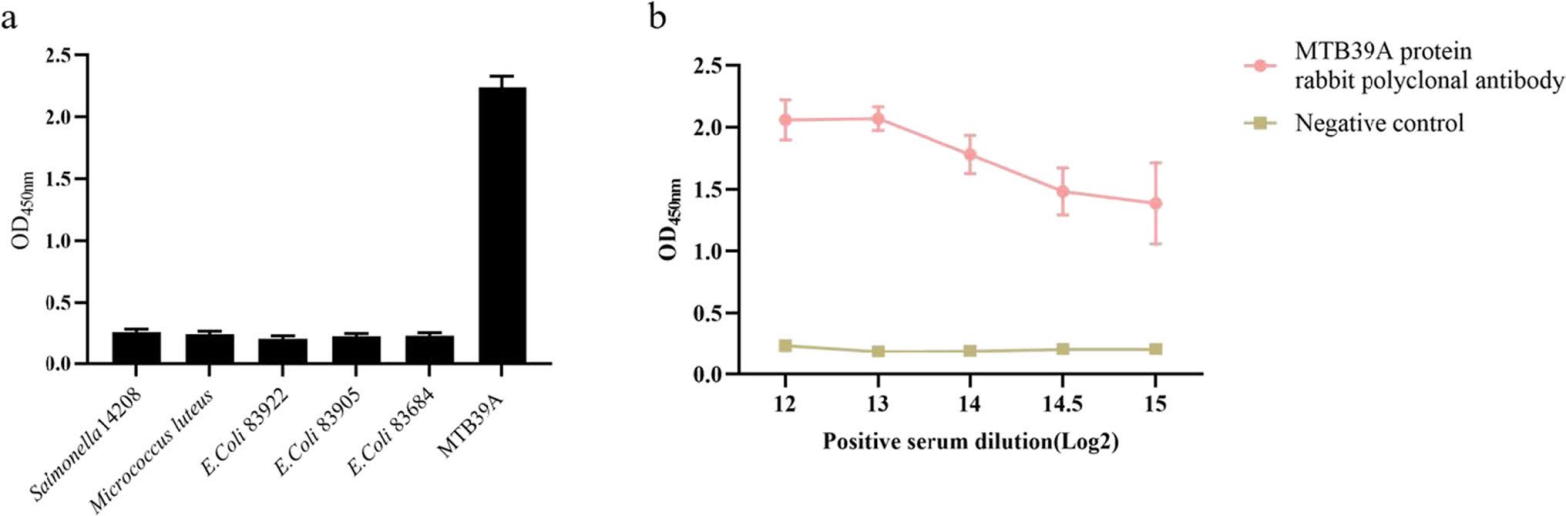
Specificity and sensitivity of rabbit polyclonal antibody to MTB39A protein. **a** ELISA results of MTB39A polyclonal antibody cross-immune reaction with common foodborne pathogens; **b** results of MTB39A polyclonal antibody sensitivity test

The specificity of the rabbit polyclonal antibody detection was verified by using MTB39A protein, *Salmonella typhimurium* 14,208, *Micrococcus garciniae*, *E. coli* 83,922, *E. coli* 83,905, and *E. coli* 83,684 cultures as the coating antigen and rabbit polyclonal antibody as the detection antibody. The results showed that the OD450 nm of the MTB39 coating antigen was 2.241, and the OD450 nm of several other bacteria were lower than the positive threshold value of 0.282, indicating that the rabbit polyclonal antibody had excellent ELISA detection specificity (Fig. 7a). The sensitivity of the established and optimized indirect ELISA was verified by serially diluting the rabbit polyclonal antibody against the MTB39A protein, and the experimental results showed that the OD450 nm was 1.3855 when the rabbit antiserum was diluted to 1:32,768, which was still above the positive threshold value of 0.282, indicating that this indirect ELISA exhibited high sensitivity (Fig. 7b).

### Stability validation of the indirect MTB39A rabbit polyclonal antibody ELISA

The established indirect ELISA was used to perform batch and interbatch assays for rabbit polyclonal and negative antibodies against the MTB39A protein, and the coefficient of variation was calculated to verify the stability of the method. The test results showed that the coefficient of variation of the 3 intrabatch assays was 1.012–2.378%, the coefficient of variation of the 3 interbatch assays was 1.100–1.825%, and the interbatch and intrabatch repeated coefficients of variation for both inter- and intrabatch replicates were less than 5%, indicating that the established method had optimal stability (Table 2).

**Table 2:** Intrabatch and interbatch stability test results of indirect ELISA

Batch	Polyclonal antibodies	Number of repetitions			Average value	Standard deviation	Coefficient of variation
		First time	Second time	Third time			
Intrabatch	MTB39A rabbit polyclonal antibody	2.088	2.047	2.075	2.070	0.020952	1.012
	Negative rabbit antibody	0.244	0.242	0.253	0.246	0.005859	2.378
Interbatch	MTB39A rabbit polyclonal antibody	2.226	2.147	2.197	2.190	0.039962	1.825
	Negative rabbit antibody	0.231	0.229	0.226	0.228	0.002517	1.100

### Preliminary application of ELISA assays for clinical samples

The established indirect ELISA method was applied to 36 human TB-positive serum samples and 21 human TB-negative serum samples. The results showed that the ELISA detected 35 human TB-positive serum samples (35/36) and 16 human TB-negative serum samples (16/21). iELISA had a sensitivity of 97.2% and a specificity of 76.1%, which indicates that this method can be initially used for the detection of clinical samples (Table 3).

**Table 3:** Testing of 57 clinical samples

Human tuberculosis positive		Human tuberculosis negative	
iELISA positive	iELISA negative	iELISA positive	iELISA negative
35	1	5	16
Sensitivity = [35/35 + 1] = 97.2% Specificity = [16/16 + 5] = 76.1% *N* = 57			

## Discussion

Currently, millions of lives are lost to TB each year due to the lack of an effective vaccine, the emergence of drug-resistant TB strains, poor diagnosis, and slow culture-based treatment evaluation. Current TB research is focused on the development of vaccines and the discovery of pathogenic markers. Markers can be used to predict the risk of *MTB* infection, the efficacy of TB treatment, vaccine efficacy, and potential antigen candidates for early diagnostic testing. Therefore, based on previous studies of *MTB* family proteins, our group screened the unique protein family of *MTB* PE/PPE proteins as potential diagnostic markers, of which PPE18 (MTB39A) was identified as being involved in the development of novel TB vaccines and the pathogenesis of the disease and is also a major component of the M72/AS01E subunit candidate vaccine (Clark et al. 2023; Harris et al. 2022). The MTB39A protein is an important member of the PE/PPE family of conserved proteins unique to *MTB* and an important virulence factor affecting the balance of Th1- and Th2-type immune responses in the host (Bhat et al. 2012; Dolasia et al. 2021). This protein causes a Th2-type immune response in anti-purified protein derivative (PPD) T cells by inducing IL-10 and IL-12 cytokines in macrophages, whereas anti-*MTB* immunity is mediated by Th1-type cellular immunity, with IL-12 regulating Th1-type cellular immunity and IL-10 interfering with Th1-type immunity. (Nair et al. 2011, 2009). MTB39A disrupts macrophage IL-12 and IL-10 balance to establish a Th2-type cellular immune response in the host that contributes to the survival of *MTB*. It is also involved in downregulating type I interferon expression (Queval et al. 2016), which blocks the macrophage response pathway to IFN-γ and leads to poor activation of the adaptive immune response in the host. These studies may help to elucidate host–pathogen interactions and facilitate the design of diagnostic methods for MTB39A to respond to *MTB* in a timely manner*.*

Polyclonal antibodies recognize multiple epitopes on antigens and have the advantages of high affinity, amplification of target proteins expressed at low levels, and the ability to recognize multiple epitopes (Gray et al. 2020; Laustsen et al. 2021). Compared to monoclonal antibodies, they are more inclusive of minor antigenic variations (e.g., polymorphism, glycosylation heterogeneity, or slight denaturation) (Mitra and Tomar 2021). Therefore, in this study, rabbit polyclonal antibodies against the MTB39A protein were prepared to achieve higher sensitivity.

Early detection is important for the prevention and control of TB, which are key for eliminating the disease. Although sputum smear microscopy is widely used, it has low sensitivity and is susceptible to interference from nontuberculous mycobacteria, and molecular diagnosis by *MTB* has a high detection rate of TB-positive specimens but can only detect approximately 50% of TB-negative specimens. Moreover, molecular diagnosis is complicated and prone to cross-contamination, which also limits its application in certain regions (Kunnath-Velayudhan et al. 2012; Mukamolova et al. 2010). In vitro isolation of *MTB* is considered the “gold standard” for the diagnosis of pulmonary and extrapulmonary tuberculosis, with a high detection rate and strain identification, but these methods have limited sensitivity and specificity, are time-consuming (6–8 weeks), and are not very useful for early diagnosis (Nambiar et al. 2017; Pai et al. 2014). The TB PPD skin test is a commonly used clinical immunological test that is used for the detection of TB due to its simplicity and speed, but it cannot clearly distinguish between BCG immunization and pathogenic *MTB* infection, has low differential diagnostic value, and is prone to high false-positive and false-negative results (Mosavari et al. 2021). In contrast, ELISA has the advantages of high sensitivity, high specificity, and suitable stability, and an indirect ELISA method using MTB39A protein as the coating antigen was established in this study (Engvall and Perlmann 1971; Nassau et al. 1976).

Here, the activity of the prepurified MTB39A protein was evaluated in human TB- and bovine TB-positive serum samples, and the obtained MTB39A protein was found to have diagnostic activity and immunoreactivity for TB. An indirect ELISA with a rabbit polyclonal antibody against the *MTB* MTB39A protein was established by immunizing New Zealand rabbits with the MTB39A protein as the immunogen and optimizing the concentration of the coating antigen (0.25, 0.5, 1, 1.5, 2, 2.5 μg/mL) and the dilution of the rabbit polyclonal antibody against MTB39A (1:2^9^, 1:2^10^…). The titer of MTB39A rabbit polyclonal antibody was 1:32,768. This indirect ELISA method has the advantages of high sensitivity, high specificity, and optimal stability for MTB39A protein detection. It has specific recognition ability for the MTB39A protein. The results of this study provide a high-quality rabbit polyclonal antibody and detection method for scientific research and rapid detection of the MTB39A protein of *MTB* and provide a good alternative detection product for better prevention and detection of *Mycobacterium tuberculosis*, which is helpful to effectively control tuberculosis infection.

## Data Availability

The original contributions presented in the study are included in the article/supplementary material, and further inquiries can be directed to the corresponding authors.

## References

[CR1] Barbier E, Fouchet T, Hartmann A, Cambau E, Mougari F, Dubois C, Lubetzki M, Rochelet M (2023). Rapid electrochemical detection of *Mycobacterium tuberculosis* in sputum by measuring Ag85 activity with disposable carbon sensors. Talanta.

[CR2] Bhat KH, Ahmed A, Kumar S, Sharma P, Mukhopadhyay S (2012). Role of PPE18 protein in intracellular survival and pathogenicity of *Mycobacterium tuberculosis* in mice. PLoS One.

[CR3] Burki T (2010). Tuberculosis-resistance, funding, and drugs. Lancet Infect Dis.

[CR4] Clark RA, Weerasuriya CK, Portnoy A, Mukandavire C, Quaife M, Bakker R, Scarponi D, Harris RC, Rade K, Mattoo SK, Tumu D, Menzies NA, White RG (2023). New tuberculosis vaccines in India: modelling the potential health and economic impacts of adolescent/adult vaccination with M72/AS01 (E) and BCG-revaccination. medRxiv.

[CR5] Correia-Neves M, Froberg G, Korshun L, Viegas S, Vaz P, Ramanlal N, Bruchfeld J, Hamasur B, Brennan P, Kallenius G (2019) Biomarkers for tuberculosis: the case for lipoarabinomannan. ERJ Open Res 5(1). 10.1183/23120541.00115-2018 10.1183/23120541.00115-2018 PMC6368998 30775376

[CR6] Dewi D, Mertaniasih NM, Soedarsono OY, Artama WT, Fihiruddin NM, Tateishi Y, Ato M, Matsumoto S (2019). Characteristic profile of antibody responses to PPD, ESAT-6, and CFP-10 of *Mycobacterium tuberculosis* in pulmonary tuberculosis suspected cases in Surabaya. Indonesia Braz J Infect Dis.

[CR7] Dolasia K, Nazar F, Mukhopadhyay S (2021). *Mycobacterium tuberculosis* PPE18 protein inhibits MHC class II antigen presentation and B cell response in mice. Eur J Immunol.

[CR8] Dosanjh DP, Bakir M, Millington KA, Soysal A, Aslan Y, Efee S, Deeks JJ, Lalvani A (2011). Novel *M tuberculosis* antigen-specific T-cells are early markers of infection and disease progression. PLoS One.

[CR9] Engvall E, Perlmann P (1971). Enzyme-linked immunosorbent assay (ELISA). Quant Assay Immunoglobulin g Immunochem.

[CR10] Gray AC, Bradbury A, Dubel S, Knappik A, Pluckthun A, Borrebaeck CAK (2020). Reproducibility: bypass animals for antibody production. Nature.

[CR11] Hakim JMC, Yang Z (2020). Predicted structural variability of *Mycobacterium tuberculosis* PPE18 protein with immunological implications among clinical strains. Front Microbiol.

[CR12] Harris RC, Quaife M, Weerasuriya C, Gomez GB, Sumner T, Bozzani F, White RG (2022). Cost-effectiveness of routine adolescent vaccination with an M72/AS01(E)-like tuberculosis vaccine in South Africa and India. Nat Commun.

[CR13] Kunnath-Velayudhan S, Davidow AL, Wang HY, Molina DM, Huynh VT, Salamon H, Pine R, Michel G, Perkins MD, Xiaowu L, Felgner PL, Flynn JL, Catanzaro A, Gennaro ML (2012). Proteome-scale antibody responses and outcome of *Mycobacterium tuberculosis* infection in nonhuman primates and in tuberculosis patients. J Infect Dis.

[CR14] Laustsen AH, Greiff V, Karatt-Vellatt A, Muyldermans S, Jenkins TP (2021). Animal immunization, in vitro display technologies, and machine learning for antibody discovery. Trends Biotechnol.

[CR15] Mitra S, Tomar PC (2021). Hybridoma technology; advancements, clinical significance, and future aspects. J Genet Eng Biotechnol.

[CR16] Mosavari N, Karimi A, Tadayon K, Shahhosseini G, Zavaran Hosseini A, Babaie M (2021). Evaluation of heating and irradiation methods for production of purified protein derivative (PPD) of *Mycobacterium tuberculosis*. Arch Razi Inst.

[CR17] Mukamolova GV, Turapov O, Malkin J, Woltmann G, Barer MR (2010). Resuscitation-promoting factors reveal an occult population of tubercle bacilli in sputum. Am J Respir Crit Care Med.

[CR18] Nair S, Ramaswamy PA, Ghosh S, Joshi DC, Pathak N, Siddiqui I, Sharma P, Hasnain SE, Mande SC, Mukhopadhyay S (2009). The PPE18 of *Mycobacterium tuberculosis* interacts with TLR2 and activates IL-10 induction in macrophage. J Immunol.

[CR19] Nair S, Pandey AD, Mukhopadhyay S (2011). The PPE18 protein of *Mycobacterium tuberculosis* inhibits NF-kappaB/rel-mediated proinflammatory cytokine production by upregulating and phosphorylating suppressor of cytokine signaling 3 protein. J Immunol.

[CR20] Nambiar R, Chatellier S, Bereksi N, van Belkum A, Singh N, Barua B, Shetty A, Rodrigues C (2017). Evaluation of mycotube, a modified version of Lowenstein-Jensen (LJ) medium, for efficient recovery of *Mycobacterium tuberculosis (MTB)*. Eur J Clin Microbiol Infect Dis.

[CR21] Nassau E, Parsons ER, Johnson GD (1976). The detection of antibodies to *Mycobacterium tuberculosis* by microplate enzyme-linked immunosorbent assay (ELISA). Tubercle.

[CR22] Ottenhoff THM (2020). A trial of M72/AS01E vaccine to prevent tuberculosis. N Engl J Med.

[CR23] Pai M, Denkinger CM, Kik SV, Rangaka MX, Zwerling A, Oxlade O, Metcalfe JZ, Cattamanchi A, Dowdy DW, Dheda K, Banaei N (2014). Gamma interferon release assays for detection of *Mycobacterium tuberculosis* infection. Clin Microbiol Rev.

[CR24] Queval CJ, Song OR, Deboosere N, Delorme V, Debrie AS, Iantomasi R, Veyron-Churlet R, Jouny S, Redhage K, Deloison G, Baulard A, Chamaillard M, Locht C, Brodin P (2016). STAT3 represses nitric oxide synthesis in human macrophages upon *Mycobacterium tuberculosis* infection. Sci Rep.

[CR25] Roos EO, Olea-Popelka F, Buss P, de Klerk-Lorist LM, Cooper D, van Helden PD, Parsons SDC, Miller MA (2018). Seroprevalence of *Mycobacterium bovis* infection in warthogs (Phacochoerus africanus) in bovine tuberculosis-endemic regions of South Africa. Transbound Emerg Dis.

[CR26] Sanchez-Cabral O, Santillan-Diaz C, Flores-Bello AP, Herrera-Ortega MI, Sandoval-Gutierrez JL, Santillan-Doherty P, Martinez-Mendoza D (2020). GeneXpert((R)) MTB/RIF assay with transbronchial lung cryobiopsy for *Mycobacterium tuberculosis* diagnosis. Ann Transl Med.

[CR27] Seele PP, Dyan B, Skepu A, Maserumule C, Sibuyi NRS (2023). Development of gold-nanoparticle-based lateral flow immunoassays for rapid detection of TB ESAT-6 and CFP-10. Biosensors.

[CR28] Tameris MD, Hatherill M, Landry BS, Scriba TJ, Snowden MA, Lockhart S, Shea JE, McClain JB, Hussey GD, Hanekom WA, Mahomed H, McShane H, Team MATS (2013). Safety and efficacy of MVA85A, a new tuberculosis vaccine, in infants previously vaccinated with BCG: a randomised, placebo-controlled phase 2b trial. Lancet.

[CR29] Ullah I, Bibi S, UlHaq I, Safia UK, Ge L, Shi X, Bin M, Niu H, Tian J, Zhu B (2020). The systematic review and meta-analysis on the immunogenicity and safety of the tuberculosis subunit vaccines M72/AS01E and MVA85A. Front Immunol.

[CR30] Wang WH, Takeuchi R, Jain SH, Jiang YH, Watanuki S, Ohtaki Y, Nakaishi K, Watabe S, Lu PL, Ito E (2020). A novel, rapid (within hours) culture-free diagnostic method for detecting live *Mycobacterium tuberculosis* with high sensitivity. EBioMedicine.

[CR31] WHO (2022, October 27) Global tuberculosis report. PUblisher. https://www.who.int/teams/global-tuberculosis-programme/data

[CR32] Zhang Q, Lu X, Gao L, Tao S, Ge Y, Cui D, Zhu R, Lu W, Wang J, Jiang S (2022). In vitro and in vivo antigen presentation and diagnosis development of recombinant overlapping peptides corresponding to *Mtb* ESAT-6/CFP-10. Front Immunol.

